# Metabolomics and transcriptomics reveal the quality formation mechanism during the processing of black tea

**DOI:** 10.1038/s41538-025-00488-7

**Published:** 2025-07-09

**Authors:** Mingjin Li, Hao Xu, Hongyu Chen, Fengjiao Ding, Qinji Li, Ziqiong Liu, Feiquan Wang, Xiaoli Jia, Yang Wu, Yun Sun, Shan Jin

**Affiliations:** 1 Key Laboratory of Ministry of education for genetics, Breeding and Multiple Utilization of Crops, College of Horticulture, Fujian Agriculture and Forestry University, 350002, Fuzhou, China; 2 Key Laboratory of Tea Science in Universities of Fujian Province, College of Horticulture, Fujian Agriculture and Forestry University, 350002, Fuzhou, China; 3 National Key Laboratory for Germplasm Innovation & Utilization of Horticultural Crops, Tea Science Department of College of Horticulture and Forestry of Huazhong Agricultural University, 430070, Wuhan, China; 4 College of Tea and Food, Wuyi University, 354300, Wuyishan, China

**Keywords:** Biochemistry, Plant sciences, Agriculture

## Abstract

The dynamic changes of metabolites and their regulatory mechanisms during black tea processing are not yet fully clear. In this study, flavonoid glycosides, tea pigments, VTs, and FADVs were primarily influenced. The content of these components continuously increased during processing, reaching their maximum after fermentation, and then decreased after drying. Withering upregulated *AM* and *GT* genes, promoting the glycosylation of flavonoids; the upregulation of *ANR* and *PPO* genes facilitated the oxidative polymerization of catechins; and the upregulation of *TPS*, *LOX*, and *HPL* genes promoted terpenoid synthesis and fatty acid degradation. This led to an increase in the content of these components in withered leaves. The accumulation of these components during fermentation was mainly due to the disruption of cells during rolling, allowing enzymes and substrates to fully integrate and react during the prolonged fermentation process. The decline in compound during the drying was primarily attributed to thermal degradation.

## Introduction

Black tea is one of the most consumed tea beverages globally, accounting for approximately 75% of worldwide tea consumption^1^. It not only possesses various health benefits such as anti-inflammatory, antibacterial, and antioxidant properties^2^, but also attracts consumers around the world due to its excellent and rich quality and flavor^3^. The processing of traditional black tea involves several steps: withering, rolling, fermentation, and drying, each playing a crucial role in the formation of its unique flavor profile. During the withering, the water loss increases the concentration of cell sap in tea leaves, enhancing the activity of various endogenous enzymes involved in the transformation of metabolites^4^. The rolling disrupts the tissue cells of the tea leaves, releasing a large number of enzymes and substrates. In the fermentation stage, these enzymes and substrates come into full contact and react, accelerating the conversion of flavor compounds^5^. These quality components undergo further transformation and recombination during the drying process, contributing to the development of the color, aroma, and taste characteristics of black tea. The intrinsic quality of tea encompasses taste, aroma, infusion color, and infused leaves. Among them, taste and aroma are the core attributes of tea quality^6,7^, which largely influence the intuitive evaluation of tea drinkers. The taste compounds in tea are primarily composed of flavonoids, organic acids, phenolic acids, amino acids, tea pigments, and carbohydrates. Meanwhile, the aroma of black tea is mainly derived from alcohols, aldehydes, esters, and ketones, with over 600 distinct aroma components having been identified^3^.

Currently, extensive research has been conducted on the dynamic changes of non-volatile^2,8,9^, volatile metabolites^3,10^ and gene expression^11,12^ during the processing of black tea. However, the majority of research has relied solely on headspace solid-phase microextraction (HS-SPME) to investigate the variation patterns of volatiles, which may not comprehensively capture all aroma compounds and could overlook some important volatiles. Additionally, the regulatory mechanisms underlying the evolution of key taste and aroma compounds during the entire processing of black tea are not fully understood. Therefore, ultra-performance liquid chromatography-quadrupole time-of-flight mass spectrometry (UPLC-QTOF-MS), push-pull dynamic headspace collection (PPDH) combined with gas chromatography-mass spectrometry (GC-MS), HS-SPME-GC-MS, and transcriptomics were employed to systematically analyze the formation and regulatory mechanisms of key taste and aroma compounds during the processing of black tea. This study lays a scientific foundation for improving the quality of black tea products in the future.

## Results and discussion

### Changes of non-volatile metabolites during processing

**Fig. 1:**
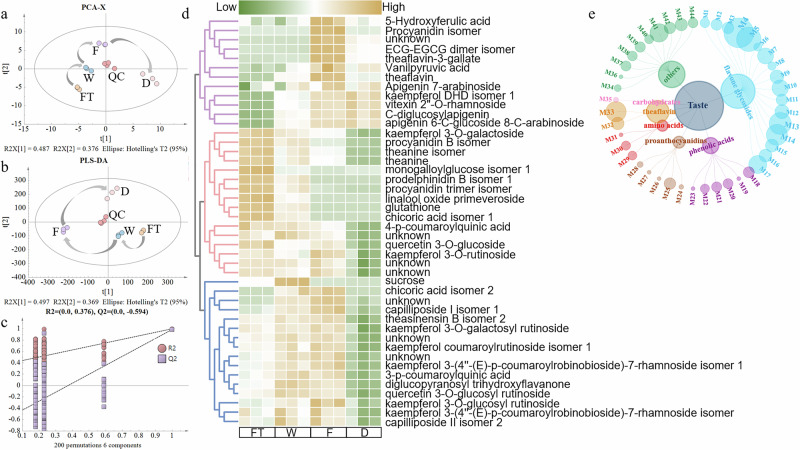
Multivariate statistical analysis. **a** Principal component analysis (PCA). **b** Partial least squares discriminant analysis (PLS-DA). **c** Permutation test. **d** Heatmap of differential metabolites. **e** Differential metabolite composition in dried finished tea, bubble size represents metabolite abundace. FT represents the sample of fresh tea leaves, W represents the sample of withered leaves, F represents the sample of fermented leaves, D represents the sample of dried finished tea, QC represents the sample of quality control. Similarly hereinafter.

To elucidate the dynamic changes of non-volatile compounds during black tea processing, non-volatile metabolites in tea leaves at various processing stages were measured using UPLC-QTOF-MS, followed by multivariate statistical analysis (Fig. 1). Correlation analysis revealed strong intra-replicate correlations (Supplementary Fig. 1), indicating high reliability and accuracy of the metabolite measurements. The principal component analysis (PCA) (Fig. 1a) clearly distinguished samples from different processing stages, suggesting differences in non-volatile metabolites among the tea samples. Additionally, the QC samples clustered near the origin, demonstrating the stability and precision of the measurement system and the reliability of the results. The partial least squares discriminant analysis (PLS-DA) results (Fig. 1b) also indicated differences in metabolites among the samples, consistent with the PCA results. The permutation test of the PLS-DA model confirmed its validity and absence of overfitting (Fig. 1c).

Based on *P* < 0.05, VIP > 1, and fold change > 2, a total of 44 differential metabolites were identified, including flavonoid glycosides, amino acids, phenolic acids, theaflavins, and proanthocyanidins (Supplementary Data 1). The clustering heatmap revealed significant changes in the levels of these differential metabolites during processing (Fig. 1d). As tea processing progressed, these differential metabolites generally showed an initial increase followed by a decrease, with fermentation being the critical stage for the synthesis of metabolites, consistent with previous research^12^. Flavonoid glycosides were the primary differential metabolites identified in this study, primarily consisting of kaempferol glycosides, which significantly contribute to the color and taste of tea infusion^6,7,13^. In this study, these flavonoid glycosides continued to increase until drying, after which they declined (Fig. 1d). This trend is largely consistent with previous research^14^. Theaflavins, crucial contributors to the infusion color of black tea, also significantly impact its flavor^8,13^. In this study, theaflavins accumulated to their maximum levels during the fermentation stage (Fig. 1d), aligning with earlier finding^8^. Besides these metabolites, others such as amino acids, proanthocyanidins, and linalool glycosides consistently decreased throughout the processing (Fig. 1d). Theanine, the most abundant amino acid in tea, has been reported to continuously decrease during black tea processing^8,10^, consistent with the results of this study (Fig. 1d). The reduction in theanine is primarily due to the interruption of its transport to new shoots after the tea leaves are harvested^8^. Additionally, the decrease in theanine may be related to the increased expression levels of *CSWRKY40* and *CSPDX2.1*. The reduction in amino acids may contribute to the formation of tea color and aroma through the Maillard reaction^15^. Proanthocyanidins, typical tea pigments, are considered bitter compounds^16^. Studies showed a declining trend of proanthocyanidins during processing^15,17^, consistent with the findings of this study (Fig. 1d).

The composition of non-volatile compounds in finished tea determines the taste quality. Thus, the differential metabolites in sample D were visualized using a network diagram (Fig. 1e; compound codes correspond to those listed in Supplementary Table 1). The diagram clearly showed that flavonoid glycosides and theaflavins had higher total abundances, indicating that high levels of these compounds were key to forming the color characteristics of black tea infusion. For instance, studies showed^6,13,18^ that higher theaflavin content resulted in a darker tea infusion color and some flavonoid glycosides were found to be significantly positively correlated with infusion color. In this study, M4 (kaempferol 3-*O*-glucosyl rutinoside) had the highest abundance, followed by M5 (quercetin 3-*O*-glucosyl rutinoside), M13 (apigenin 7-arabinoside), and M14 (capilliposide I isomer 1). One study showed^13^ that the abundance of kaempferol 3-*O*-glucosyl rutinoside was significantly negatively correlated with the color of beauty tea infusion and positively contributes to the sweetness and mellowness, while capilliposide I isomer 1 shows the opposite effect. Quercetin 3-*O*-glucosyl rutinoside was also considered one of important contributors to the infusion color and taste of Qingxiang Tieguanyin tea^7^.

### The accumulation of flavonoid glycosides enhances both the flavor and health benefits of tea

Flavonoids predominantly exist in plants in the form of glycosides, primarily formed through oxidation (including hydroxylation and methoxylation) at key positions on the flavonoid backbone, exhibiting significant biological potential for antioxidant, hepatoprotective, anti-inflammatory, anticancer, and antiviral activities^19^. Sugar substituents are typically linked to the flavonoid carbon skeleton in hydroxylated forms, forming *O*-glycosides, or directly connected to the *C* atoms on the flavonoid *A*-ring to form *C*-glycosides. Flavonols such as myricetin, kaempferol, and quercetin are the most widely distributed flavonoids, and their glycosides generally exist as *O*-glycosides, with the glycosyl group primarily attached at the *C*-3 position, a conclusion supported by our research findings (Supplementary Data 1).

From a flavor perspective, flavonoid glycosides have a velvety, silky-astringent, and mouth-coating sensation^20^. Flavonoid glycosides have very low taste thresholds, making them essential contributors to the taste quality of tea. For instance, the threshold of quercetin-3-*O*-[*α*-l-rhamnopyranosyl-(1 → 6)-*O*-*β*-*D*-glucopyranoside], quercetin 3-*O*-rutinoside, and kaempferol 3-*O*-rutinoside are as low as 0.001, 0.00115, and 0.25 μmol/L, respectively, which is far lower than for EGCG (190 μmol/L) and ECG (260 μmol/L)^19^. Flavonoid glycosides are hydrolytically stable, enhancing their in vitro absorption^21^. From a nutritional and dietary perspective, the substantial accumulation of flavonoid glycosides results in higher concentrations of bioactive compounds in brewed tea, enhancing its health benefits and contributing to a mellower, thicker, and more harmonious taste^19^.

Previously, it was believed that the enhancement of tea flavor was closely related to the increase in catechins^22,23^, and that a reduction in catechins could decrease the bitterness and astringency of tea infusion^24^, significantly improving tea flavor. Based on metabolomic analysis and the role of these compounds in food flavor and health, we propose that the accumulation of flavonoid glycosides, rather than changes in catechin content, can enhance tea flavor. Given the importance of *O*-glycosylflavonoids in stress resistance and human health, further in-depth research is needed.

### Changes in volatiles monitored in real-time during processing

**Fig. 2:**
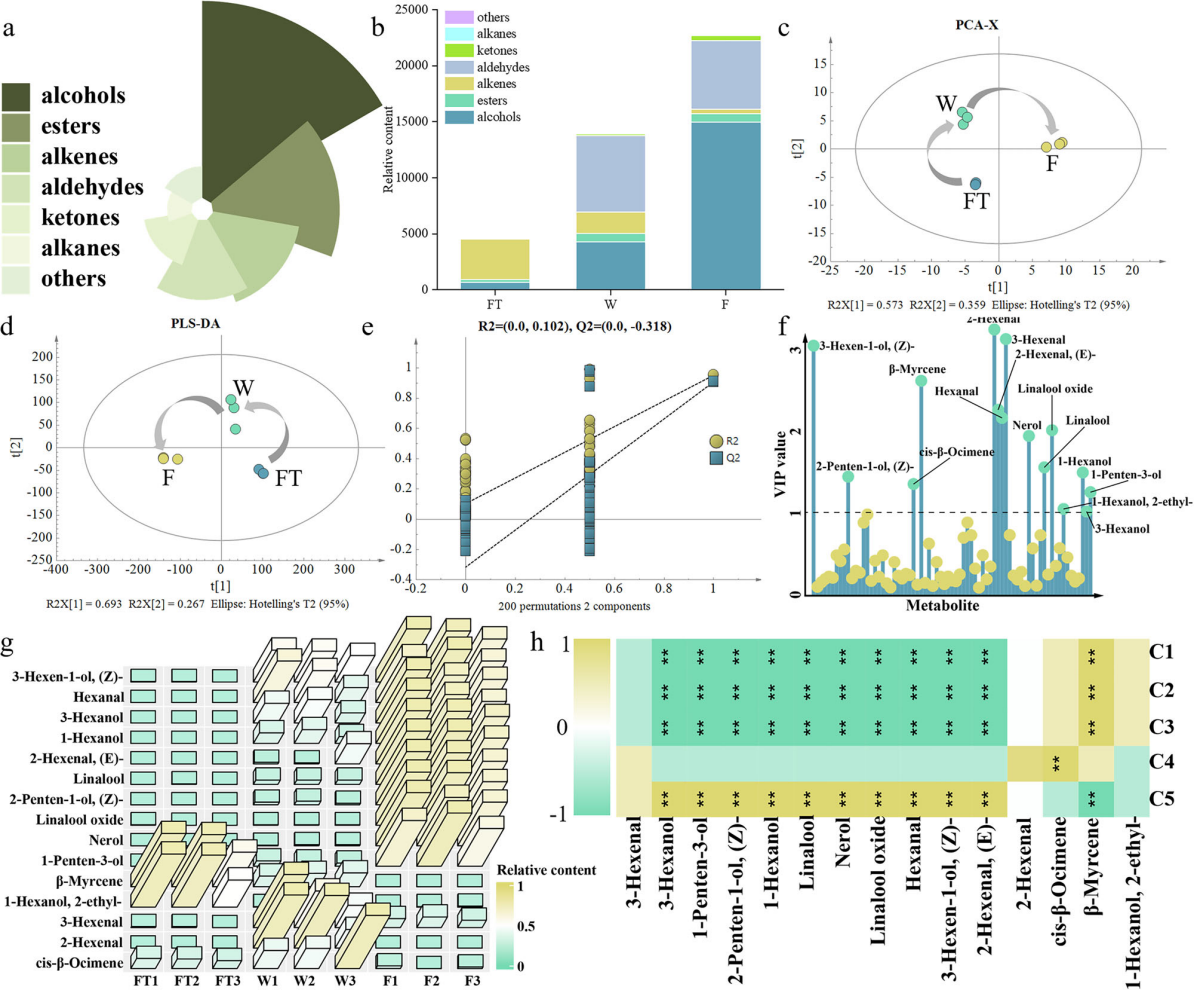
Multivariate statistical analysis. **a** The proportion of volatiles. **b** Stacked bar chart of volatiles. **c** PCA. **d** PLS-DA. **e** Permutation test. **f** Variable importance in projection (VIP) analysis. **g** Heatmap of key volatiles. **h** Heatmap of correlation between non-volatile metabolites and key volatiles.

To monitor the dynamic changes of volatiles in tea leaves during processing in real-time, the PPDH method combined with GC-MS was used to measure the volatiles. A total of 73 volatiles were identified (Supplementary Data 2; no volatiles were detected in sample D). As processing progressed, the number of volatiles increased (27 volatiles in FT, 56 in W, and 59 in F). These volatiles primarily included alcohols, esters, alkenes, and aldehydes (Fig. 2a), consistent with previous research^10^. Additionally, the total content of volatiles also continuously increased. Among them, alcohols and ketones increased throughout the processing, while alkenes showed the opposite trend. Aldehydes and esters initially increased and then slightly decreased, whereas other compounds such as alkanes and aromatic compounds showed little change during processing (Fig. 2b). These results indicate that aldehydes are mainly formed during the withering stage, and fermentation is the critical step for the accumulation of alcohols. It has been reported that the primary aroma compounds of black tea are alcohols and aldehydes^25^, which aligns with the findings of this study.

The PCA (Fig. 2c) and PLS-DA (Fig. 2d) showed clear separation of tea samples from different processing stages, indicating differences in volatiles among the samples. The permutation test of the PLS-DA confirmed its validity and absence of overfitting (Fig. 2e). Based on the variable importance in projection (VIP) analysis from PLS-DA, a total of 15 volatiles with VIP > 1 exhibited significant changes during processing (Fig. 2f), primarily consisting of fatty acid derivatives (FADVs) and volatile terpenes (VTs). As shown in Fig. 2g, these key volatiles showed an increasing trend as processing progressed, with most of them sharply increasing during the fermentation stage, suggesting that fermentation significantly promotes fatty acid degradation and terpenoid synthesis. In summary, the PPDH analysis results indicate that processing primarily promotes fatty acid degradation and terpenoid synthesis, leading to the substantial generation of aldehydes and alcohols during processing, which become the main aroma components. This also drives the continuous increase in the number and total content of volatiles up to the drying stage.

Additionally, a correlation analysis was conducted between the representative non-volatile compounds and these 15 key volatiles. As shown in Fig. 2h, linalool oxide primeveroside (C1), theanine (C2), and glutathione (C3) exhibited highly significant negative correlations (*P* < 0.01) with 10 key volatiles and a highly significant positive correlation (*P* < 0.01) with *β*-myrcene. In contrast, kaempferol 3-*O*-glucosyl rutinoside (C5) showed the opposite trend. Sucrose (C4) showed a highly significant positive correlation (*P* < 0.01) with *cis*-*β*-ocimene but no significant correlations with other compounds. During tea processing, particularly under the action of rolling, the damaged tea tissues release enzymes that hydrolyze glycosidic bonds. This releases monoterpenols such as linalool and its oxides, and geraniol^26^. Furthermore, the aglycones produced during hydrolysis form *O*-flavonoid glycosides with flavonols through hydroxylation. This may explain the highly significant positive correlation between flavonoid glycosides and linalool and its oxides, as well as the highly significant negative correlation between linalool oxide primeveroside and linalool oxide (Fig. 2h).

### Changes in volatiles in freeze-dried samples during processing

**Fig. 3:**
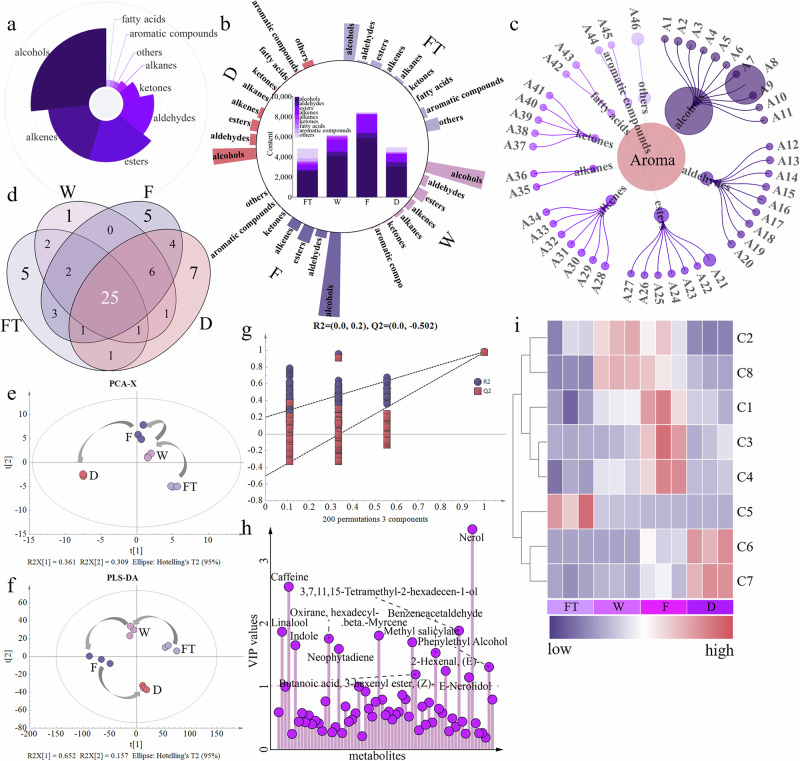
Multivariate statistical analysis. **a** The proportion of volatiles. b Bar chart of various volatiles and stacked bar chart of total volatiles. **c** Volatile metabolite compostion in dried finished tea, bubble size represents the content of volatile metabolite. **d** Venn diagram. **e** PCA. **f** PLS-DA. **g** Permutation test. **h** VIP analysis, **i** Heatmap of characteristic volatiles.

To elucidate the dynamic changes of volatiles in freeze-dried samples during processing, HS-SPME coupled with GC-MS was used to measure the volatiles. A total of 64 volatiles were identified (Supplementary Data 3), primarily including alcohols, alkenes, esters, and aldehydes (Fig. 3a). As processing progressed, the total content of volatiles in the tea leaves showed an initial increase followed by a decrease (Fig. 3b). These results were consistent with the aroma analysis obtained using the PPDH method. Alcohols, aldehydes, esters, and alkenes all increased during processing and peaked after fermentation (Fig. 3b). The aroma characteristics of the finished tea are determined by the composition of its volatiles. To provide a more intuitive understanding of the volatile composition in the finished tea, the volatiles in sample D were visualized using a network diagram (Fig. 3c; with volatile codes corresponding to those listed in Supplementary Table 2). Among them, alcohols were the main aroma components, followed by aldehydes and esters. The content of A8 (nerol) was higher than that of other volatiles, while the contents of the remaining volatiles were relatively similar (Fig. 3c). Venn diagram results showed that there were 25 common volatiles among these tea samples, and the number of unique volatiles formed at each stage increased as processing progressed (Fig. 3d). PCA and PLS-DA results (Fig. 3e, f) revealed clear separation of tea samples from different processing stages, indicating differences in volatiles among the freeze-dried samples. The permutation test of the PLS-DA confirmed its validity and absence of overfitting (Fig. 3g). Additionally, based on VIP > 1, a total of 14 volatiles exhibiting significant changes during processing were identified (Fig. 3h). Unlike the results from the PPDH method, these key volatiles were primarily VTs and amino acid derivatives (AADVs), with fewer FADVs.

The final contribution of volatiles to the aroma quality of tea depends on their odor activity value (OAV)^16^. To further understand the characteristic volatiles that contribute to the formation of aroma quality during black tea processing, the OAVs of the volatiles were calculated and 8 characteristic volatiles were identified (VIP > 1 & OAVs ≥ 1). Linalool, nerol, and benzeneacetaldehyde were identified as the primary aroma contributors. The remaining volatiles, such as *β*-myrcene, methyl salicylate, indole, (*E*)-2-hexenal, and phenylethyl alcohol, also significantly contributed to the aroma characteristics of the tea during processing (Supplementary Table 3). Compared to FT, the contents of the 7 characteristic volatiles, except for C5 (indole), increased during processing. The levels of C6 (benzeneacetaldehyde) and C7 (phenylethyl alcohol) peaked after drying. The contents of C1 (nerol), C3 (*β*-myrcene), and C4 (methyl salicylate) peaked after fermentation, while the contents of C2 (linalool) and C8 ((*E*)-2-hexenal) were highest after withering and fermentation (Fig. 3i). Among the numerous volatiles, phenylethyl alcohol, benzeneacetaldehyde, (*E*)-2-hexenal, linalool, and methyl salicylate have been identified as key aroma-active compounds in black tea^5^, consistent with the findings of this study. Additionally, *β*-myrcene, linalool, and methyl salicylate were also considered key aroma-active substances in beauty tea made from different fresh leaf materials^27^. Although these characteristic volatiles are decisive for the aroma quality of black tea, the potential additive effects of other volatiles (OAVs < 1), such as synergistic or antagonistic effects, should not be overlooked. In summary, the HS-SPME analysis results indicate that processing primarily promotes terpenoid synthesis and amino acid degradation, leading to the continuous increase of alcohols, aldehydes, and esters during processing, peaking after fermentation, thereby driving the increase in the total content of volatiles. Furthermore, 8 characteristic volatiles, including linalool, nerol, and *β*-myrcene, were identified, with phenylalanine-derived products being largely produced during the drying stage, while glycoside-derived products were mainly formed during the fermentation stage.

### Transcriptomic analysis

After removing low-quality reads, ambiguous reads, and adapter sequences, each library contained 22.21-25.00 million clean reads (Supplementary Table 4). These clean reads were mapped to the tea reference genome, with the alignment efficiency of reads to the reference genome ranging from 87.61% to 95.06% across samples (Supplementary Table 5). Additionally, 24,425 genes predicted from the genome were expressed in at least one sample (Supplementary Data 4). Furthermore, 4405 new genes, not included in the reference genome, were identified. Ten genes were selected for quantitative real-time polymerase chain reaction (qRT-PCR) validation. The results showed that the relative expression levels were consistent with the RNA-Seq data (Supplementary Fig. 2), supporting the accuracy and reliability of the transcriptomic analysis results.

**Fig. 4:**
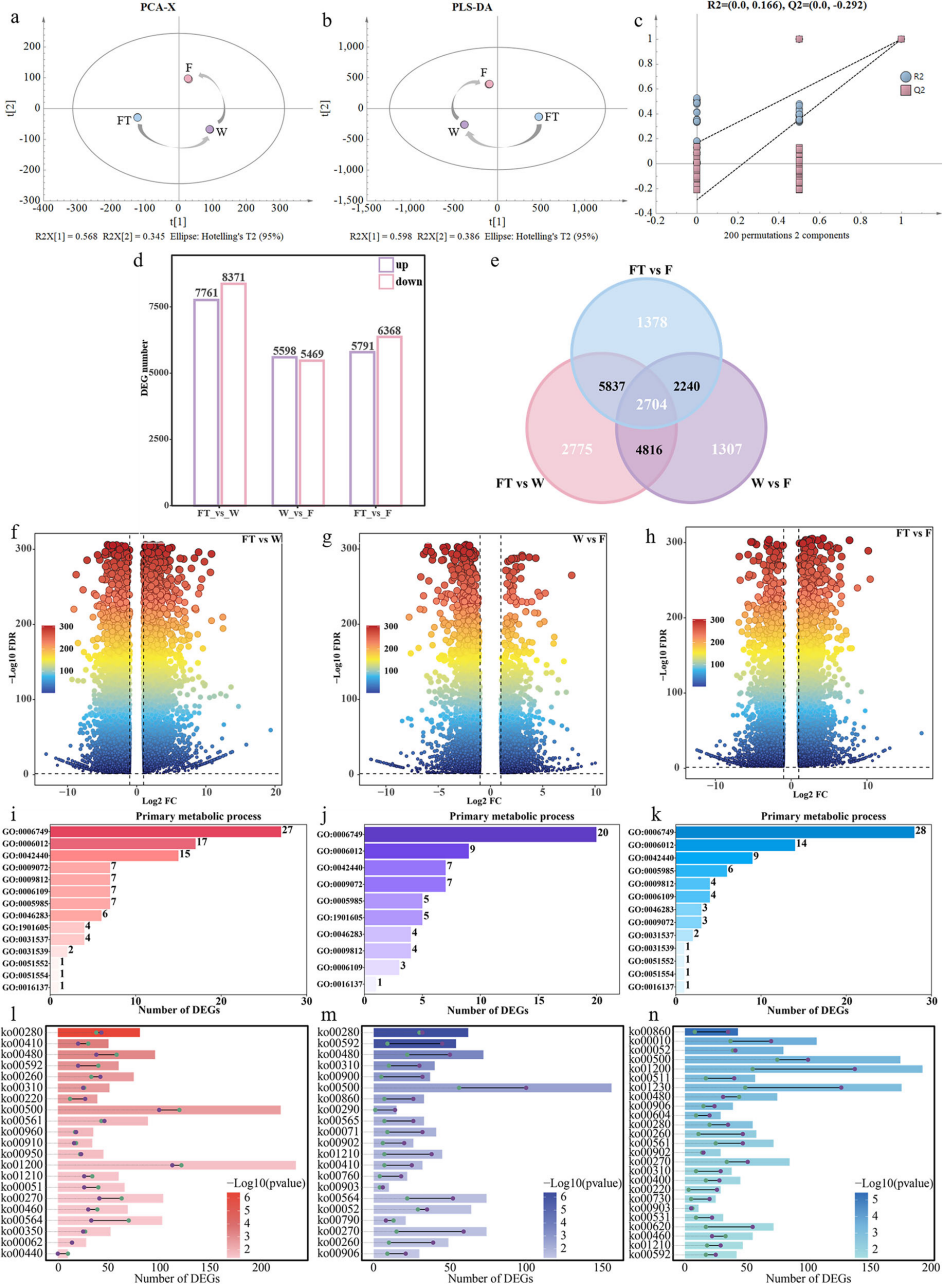
Multivariate statistical analysis and enrichment analysis of differential expressed genes (DEGs). **a** PCA. **b** PLS-DA. **c** Permutation test. **d** Bar chart of DEGs. **e** Venn plot. **f**–**h** Volcano plot of the FT vs W group, W vs F group, and FT vs F group, respectively. **i**–**k** Genetic ontological (GO) enricment analysis of the FT vs W group, W vs F group, and FT vs F group, respectively. **l**–**n** Kyoto encyclopedia of genes and genomes (KEGG) pathway enrichment analysis, green dots represent upregulated genes, while purple dots represent downregulated genes.

Multivariate statistical analysis showed differences in gene expression among tea samples at different processing stages, with the total explained variance exceeding 91%. Additionally, PCA (Fig. 4a) and PLS-DA (Fig. 4b) showed low variability between biological replicates, and the correlation heatmap also indicated strong correlations among replicates (Supplementary Fig. 3), demonstrating high reproducibility in gene expression patterns. The permutation test of the PLS-DA model confirmed its validity (Fig. 4c). Through differential expression analysis across the three processing stages, 21,057 differentially expressed genes (DEGs) were identified. Among them, 16,132 DEGs were identified in the FT vs W comparison, including 7761 upregulated and 8371 downregulated genes (Fig. 4d, f), suggesting that downregulated genes may play a more significant role during the withering stage of black tea processing. In the W vs F comparison, 11,067 DEGs were identified, including 5598 upregulated and 5469 downregulated genes (Fig. 4d, g), indicating that upregulated genes may play a more important role during the fermentation stage of black tea processing. In the FT vs F comparison, 12,159 DEGs were identified, including 5791 upregulated and 6368 downregulated genes (Fig. 4d, h). Among the three comparison groups, 2704 common DEGs and 5460 unique DEGs were identified (Fig. 4e). Among the 2704 common DEGs, genes involved in metabolic processes within biological processes played a dominant role (Supplementary Data 5), indicating significant changes in metabolites in tea leaves in response to abiotic stress during processing. The remaining 18,353 DEGs accounted for 87.16% of the total DEGs, suggesting a broad range of genetic variation among tea samples at different processing stages.

As shown in Fig. 4i–k, Genetic ontological (GO) enrichment analysis revealed that glutathione metabolic process (GO:0006749), galactose metabolic process (GO:0006012), and sucrose metabolic process (GO:0005985) were the primary metabolic processes. Kyoto encyclopedia of genes and genomes (KEGG) pathway enrichment analysis was also conducted. As shown in Fig. 4l–n, the DEGs in all three groups were significantly enriched in amino acid metabolism pathways (*P* < 0.05), followed by fatty acid and carbohydrate metabolism pathways. In the FT vs W group, ko00280 (Valine, leucine, and isoleucine degradation), ko00410 (*beta*-Alanine metabolism), and ko00480 (Glutathione metabolism) were the most significant (*P* < 0.001), with most DEGs in these pathways being upregulated. In the W vs F group, ko00280 (Valine, leucine, and isoleucine degradation), ko00592 (*α*-Linolenic acid metabolism), and ko00480 (Glutathione metabolism) were the most significant (*P* < 0.001), with most DEGs in these pathways being downregulated. However, in the FT vs F group, carbohydrate metabolism pathways were the most significant, and most DEGs in these pathways were also downregulated. In summary, amino acid, fatty acid, and carbohydrate metabolism were the most prominent during tea processing, and the expression levels of most genes involved in these metabolic pathways decreased during processing.

### Processing enhances the content of flavonoid glycosides by promoting the glycosylation of flavonoids

**Fig. 5:**
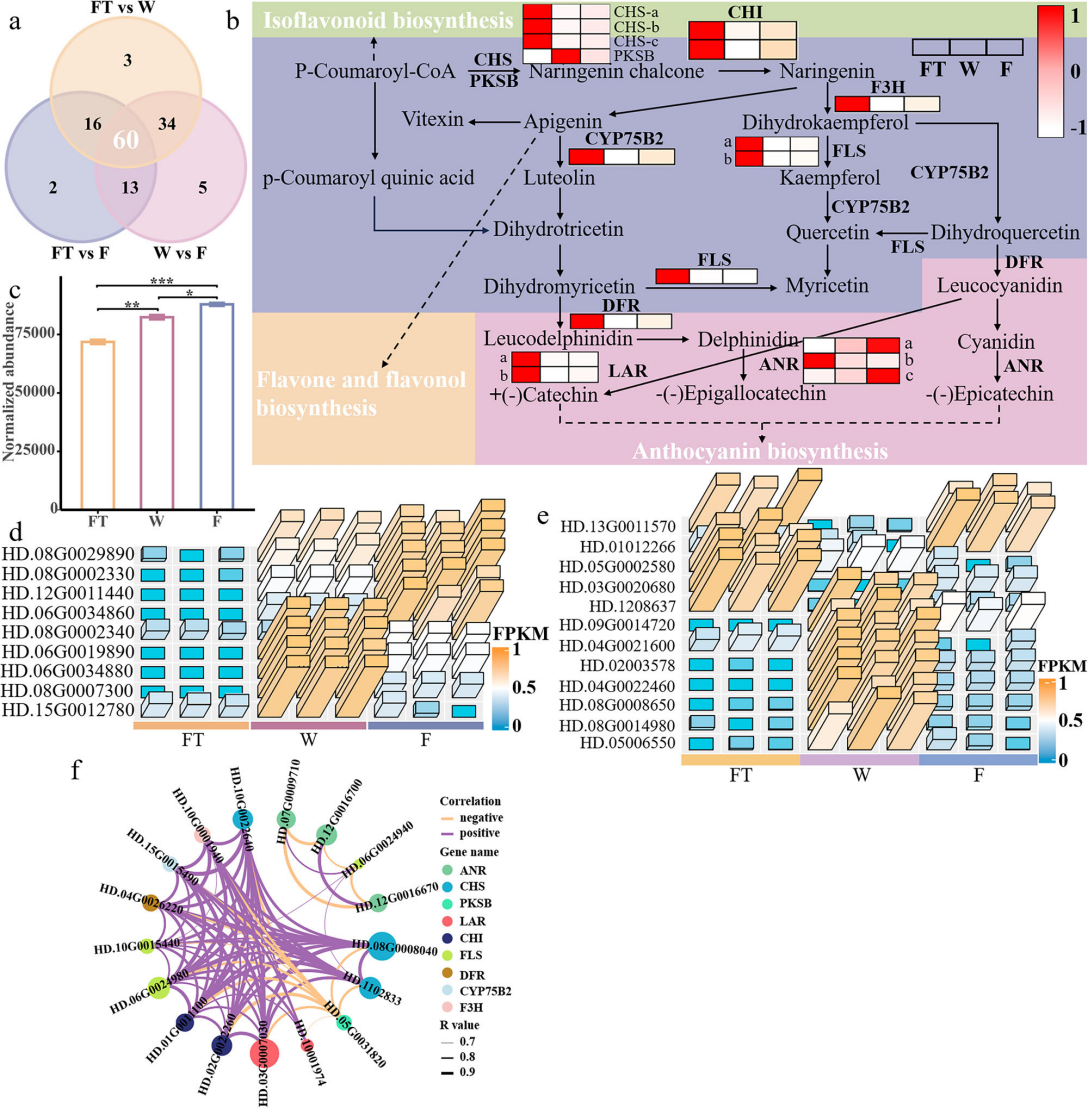
The regulatory mechanism of the flavonoid biosynthesis pathway. **a** Venn plot. **b** Flavonoid metabolic pathway. **c** Flavonoid glycosides abundance in tea at each processing stage. “*” indicates significant difference (*P* < 0.05), “**” indicates highly significant difference (*P* < 0.01), “***” indicates extremely significant difference (*P* < 0.001). **d** Heatmap of amylase (AM) gene expression. **e** Heatmap of glycosyltransferase (GT) gene expression. **f** Correlation between genes of related enzymes in flavonoid metabolism pathway. CHS Chalcone synthase, CHI Chalcone isomerase, F3H Flavanone 3-hydroxylase, FLS Flavonol synthase, DFR Dihydroflavonol 4-reductase, LAR Leucoanthocyanidin reductase, ANR Anthocyanidin reductase, PKSB Type III polyketide synthase B, CYP75B2 Flavonoid 3’-monooxygenase.

In this study, flavonoid glycosides were the main differential metabolites. Given the notable changes in flavonoid glycosides and their importance to flavor and infusion color quality, the regulatory mechanisms of flavonoid glycosides during processing were emphasized. A total of 60 genes related to the differential accumulation of flavonoids during black tea processing were identified (Fig. 5a, Supplementary Data 6) and a flavonoid metabolic pathway was further constructed. As shown in Fig. 5b, most enzyme genes involved in flavonoid biosynthesis were downregulated during processing. This is because gene regulation primarily occurs before harvesting, and the expression of most genes rapidly decreases after fresh leaves are plucked^2^. It has been reported that the expression of chalcone synthase (*CHS*), chalcone isomerase (*CHI*), flavanone 3-hydroxylase (*F3H*), dihydroflavonol 4-reductase (*DFR*), leucoanthocyanidin reductase (*LAR*), anthocyanidin reductase (*ANR*), and flavonol synthase (*FLS*) in Wuyi black tea significantly decreased during processing^2^, further supporting our results. Interestingly, the total abundance of flavonoid glycosides in this study continuously increased as processing progressed (Fig. 5c), indicating that the accumulation of flavonoid glycosides is mainly regulated by other key enzyme genes. It has been reported that the accumulation of flavonoid glycosides is associated with the glycosylation of flavonoids^14^. Therefore, several amylase (*AM*) and glycosyltransferase (*GT*) genes were identified (Supplementary Data 7 and 8). As shown in Fig. 5d, among these highly expressed AM genes, their expression levels in FT were significantly lower than in W and F (*P* < 0.05). This leads to the production of more monosaccharides during the withering and fermentation. These monosaccharides can easily form flavonoid glycosides with flavonoids (such as kaempferol and quercetin) under the action of *GTs*^14^. The expression levels of most *GT* genes were higher in W and lower in F (Fig. 5e). During withering, prolonged water loss stress activates *AMs* and *GTs*. Simultaneously, cell membrane permeability increases, and the concentrations of enzymes and substrates rise^8^. This results in a highly significant increase in the content of flavonoid glycosides in W (*P* < 0.01, Fig. 5c). Rolling disrupts the tissue cells of tea leaves, further enhancing the contact between enzymes and substrates during fermentation and making the related enzymatic reactions more thorough. However, since the expression levels of *GT* genes decreased during the fermentation (Fig. 5e), this may explain why the rate of flavonoid glycoside generation was lower during fermentation than during withering, although the overall content of flavonoid glycosides still increased (Fig. 5c), consistent with previous research^2,10^. In summary, withering enhances the expression of *AM* and *GT* genes, while fermentation increases the expression of *AM* genes and, through the action of rolling, significantly enhances the contact between relevant enzymes and substrates. This provides ample substrates for the glycosylation of flavonoids, catalyzed by *GTs*, leading to the continuous accumulation of flavonoid glycosides during processing.

### Processing enhances the content of tea pigments by increasing the expression of *ANR* and *PPO* genes

Theaflavins are typical tea pigments in black tea. In this study, the abundance of theaflavins increased during processing and peaked during the fermentation stage (Fig. 1d), consistent with previous research^2^. *ANR* is an enzyme at the end of the flavonoid metabolic pathway, involved in the formation of epicatechin in tea plants, which is a precursor for the synthesis of theaflavins. In this study, the expression levels of some *ANR* genes showed an increasing trend during processing (Fig. 5b), aligning with the trend of theaflavin changes. The upregulation of *ANR* promoted the accumulation of epicatechin in tea leaves, providing more substrates for theaflavin formation. Catechins can be oxidatively polymerized to form theaflavins under the action of polyphenol oxidase (*PPO*) and peroxidase (*POD*)^14^. Therefore, two *PPO* genes were identified and found that their expression levels initially increased and then decreased during processing, with the lowest expression in FT (Supplementary Fig. 4), consistent with previous research^2^. Dehydration during withering not only enhances the concentration of enzymes and substrates but also increases the expression of *ANR* and *PPO* genes. This accelerates the oxidative polymerization of catechins and promotes the accumulation of theaflavins. During fermentation, the expression of *ANR* genes further increased, strengthening the generation of epicatechin. Although the expression of *PPO* genes decreased, the disruption of tea leaf cells caused by rolling enhanced the interaction between enzymes and substrates^28^, leading to more thorough enzymatic oxidation of catechins during the prolonged fermentation process, thereby increasing the content of theaflavins. In summary, the high expression of *ANR* and *PPO* during processing, as well as the sufficient integration and reaction between the enzymes and substrates, promote the generation and accumulation of theaflavins.

Proanthocyanidins are also typical tea pigments. In this study, some proanthocyanidins, such as procyanidin isomer and ECG-EGCG dimer isomer, showed trends similar to those of theaflavins (Fig. 1d). Given that proanthocyanidins are also formed through the oxidative polymerization of catechins, we believe that the accumulation of these two proanthocyanidins is regulated by the expression of *ANR* and *PPO* genes. Previous studies found^9,10,17^ that the content of proanthocyanidins continuously decreased during processing. In this study, the abundance of some proanthocyanidins, such as procyanidin B isomer, prodelphinidin B isomer, and procyanidin trimer isomer 1, also decreased during processing (Fig. 1d), indicating that the changes in these proanthocyanidins are primarily influenced by other key enzyme genes. One study showed that the *ANS* gene played an important role in the formation of ECG and EGCG, and the downregulation of this gene led to a reduction in catechins and proanthocyanidins^19^. In this study, the *DFR* gene upstream of cyanidin and delphinidin was significantly downregulated during the withering and fermentation stages (*P* < 0.05), and it was speculated that the downregulation of the *DFR* gene contributed to the reduction of some proanthocyanidins. Additionally, enzymatic and spontaneous oxidation of proanthocyanidins may be potential factors in their decreased content^14^. In summary, the activation of *ANR* and *PPO* during processing promotes the generation of some proanthocyanidins, while the reduction of others is related to the inhibition of key enzymes such as *DFR*.

Additionally, the R package was employed to perform correlation analysis (*P* < 0.05 & |r | > 0.7) on the enzyme genes in the flavonoid biosynthesis pathway (Fig. 5f). The results showed that these genes were significantly correlated (*P* < 0.05) and primarily exhibited positive correlations, indicating that the expression of gene groups within the same metabolic pathway tends to be similar.

### Regulation mechanism of key volatiles during processing

Based on their precursors, the key volatiles identified by the two extraction methods are primarily classified into VTs, FADVs, and AADVs.

**Fig. 6:**
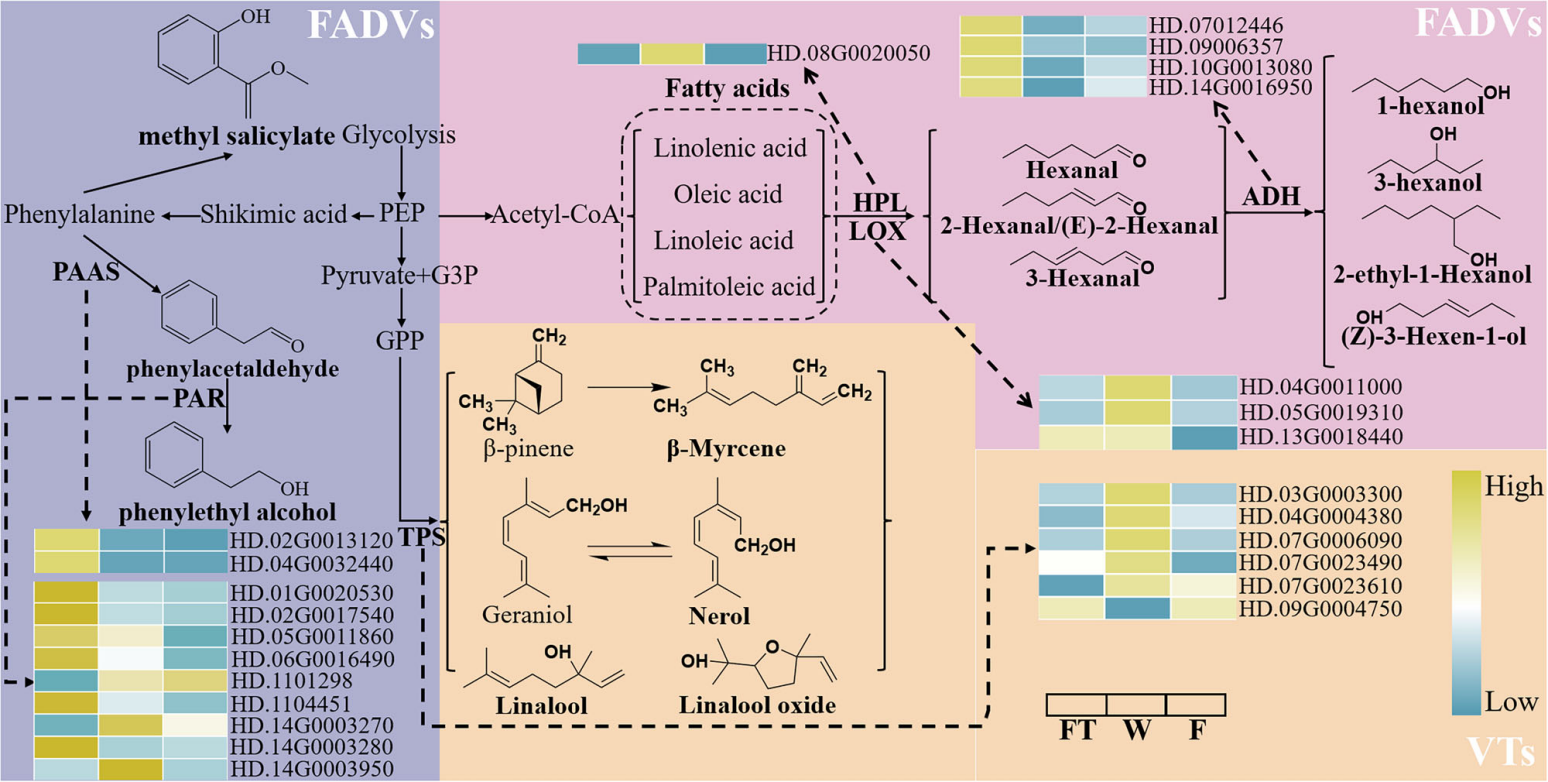
Metabolic pathways of key volatiles. HPL Hydroperoxide lyase, LOX Lipoxygenases, ADH Alcohol dehydrogenase, PAAS Phenylacetaldehyde synthase, PAR Phenylacetaldehyde reductase, TPS Terpene synthase.

VTs: The analysis results of both extraction methods indicated that the content of VTs increased during processing and peaked during the fermentation stage. VTs originate from the monoterpene biosynthesis pathway and are catalyzed by terpene synthases (*TPSs*) (Fig. 6). The results of this study showed that among the highly expressed *TPS* genes (Supplementary Data 9), their expression was upregulated during the withering stage (Fig. 6). This suggests that dehydration stress readily activates *TPS*, promoting the synthesis of VTs. However, the expression of *TPS* genes decreased during the fermentation stage (Fig. 6), which did not reduce the generation and release of VTs. This is because, during the rolling process, tissue cells are disrupted, releasing a large number of enzymes and substrates, which further fully integrate and react in the prolonged warm and humid environment, thus further promoting the generation and accumulation of VTs during fermentation. Notably, the level of VTs decreased during the drying process, which may be attributed to the recombination of VTs with glycosides to form non-volatile compounds in the early stages of drying. Subsequently, prolonged high temperatures deactivated the enzymes, causing extensive decomposition due to their high thermal sensitivity^29^. In summary, withering activates *TPS*, while rolling and fermentation enhance the integration and reaction of enzymes and substrates, thereby continuously generating and accumulating VTs before drying.

FADVs: Similar to VTs, the levels of FADVs increased during processing and peaked during the fermentation stage. These FADVs are derived from the oxidative degradation of unsaturated fatty acids, a pathway that is a major route for the formation of tea flavor^26^. Fatty acids are first oxidized by lipoxygenases (*LOXs*) into lipid hydroperoxides, which are then cleaved by hydroperoxide lyases (*HPLs*) into six-carbon aliphatic aroma compounds such as hexanal. Subsequently, these aldehydes can be further reduced to their corresponding alcohols or isomerized into trans-isomers and then reduced to alcohols by alcohol dehydrogenases (*ADHs*)^26^. In this study, several *LOX*, *HPL*, and *ADH* genes were identified (Supplementary Data 10-12). Among the highly expressed genes, the expression of *LOX* and *HPL* genes was upregulated during the withering stage (Fig. 6). This suggests that dehydration stress readily activates *LOX* and *HPL*, thereby promoting the synthesis of aldehyde fatty acid derivatives. The expression of *LOX* and *HPL* genes decreased during the fermentation stage (Fig. 6), which may explain the decline in the content of 2-hexanal, 3-hexanal, and (*E*)-2-hexanal. Compared to FT, the expression of ADH genes was downregulated during both the withering and fermentation stages (Fig. 6), which may explain the decrease in 2-ethyl-1-hexanol levels. However, the content of other alcohol fatty acid derivatives was highest during the fermentation stage. This indicates that alcohol fatty acid derivatives are primarily influenced by other factors. Continuous dehydration during withering increases the concentration of enzymes and substrates, promoting related enzymatic reactions^8^; rolling and fermentation further enhance the integration of relevant enzymes and substrates, thereby facilitating the reduction reactions catalyzed by *ADH*. This may be the main reason for the increase in the content of alcohol fatty acid derivatives and also explains why the downregulation of *LOX* and *HPL* genes during the fermentation stage did not lead to a decrease in the content of hexanal and (*E*)-2-hexanal. Similarly, the reduction in FADVs content during the drying process may be attributed to the thermal degradation of volatiles. In summary, withering activates *LOX* and *HPL* and increases the concentration of related enzymes and substrates, thereby promoting the synthesis of FADVs, while the further accumulation of FADVs during the fermentation stage is mainly due to the thorough reaction of enzymes and substrates.

AADVs: The contents of benzeneacetaldehyde and phenylethyl alcohol increased during processing and peaked during the drying stage (Fig. 3i). Benzeneacetaldehyde can be directly derived from phenylalanine^3^. During tea processing, phenylalanine can be converted into benzeneacetaldehyde under the catalysis of phenylacetaldehyde synthase (*PAAS*). Benzeneacetaldehyde can be further transformed into phenylethyl alcohol by phenylacetaldehyde reductase (*PAR*), while phenylethyl alcohol can be oxidized back to benzeneacetaldehyde^3^. This explains why the trends of benzeneacetaldehyde and phenylethyl alcohol are consistent (Fig. 3i). Interestingly, in this study, the expression of *PAAS* (Supplementary Data 13) and *PAR* genes (Supplementary Data 14) decreased during processing (Fig. 6), indicating that benzeneacetaldehyde and phenylethyl alcohol primarily originate from other pathways. In addition to enzymatic synthesis, the Strecker degradation of amino acids is considered the main reason for the increase in benzeneacetaldehyde content. High temperatures increase the content of phenylethyl alcohol by promoting the Strecker reaction of amino acids and the Maillard reaction between amino acids and sugars. In summary, the increase in benzeneacetaldehyde and phenylethyl alcohol content during processing is mainly attributed to high temperatures promoting the Strecker reaction of amino acids and the Maillard reaction between amino acids and sugars.

In summary, the processing of black tea mainly affected flavonoid glycosides, tea pigments, VTs, and FADVs. The content of these components continuously increased during processing, reaching their maximum during the fermentation stage, and then rapidly decreased during the drying stage. Withering activated *AM* and *GT*, promoting the glycosylation of flavonoids, thereby leading to the accumulation of flavonoid glycosides. The activation of *ANR* and *PPO* facilitated the oxidative polymerization of catechins, resulting in the accumulation of tea pigments. The activation of *TPS* promoted the generation of VTs, while the activation of *LOX* and *HPL* enhanced the synthesis of FADVs. The accumulation of these components during fermentation was mainly due to the disruption of cells during rolling, allowing enzymes and substrates to fully integrate and react during the prolonged fermentation process. During the drying stage, prolonged high temperatures deactivated the enzymes, causing thermal degradation of these quality components and leading to a rapid decline in their content. Furthermore, eight characteristic volatiles, including linalool, nerol, and benzeneacetaldehyde, are the main contributors to the aroma during black tea processing. However, the key enzymes and regulatory networks involved in the changes of these critical quality components during black tea processing have not yet been thoroughly investigated. In the future, integrating multi-omics approaches such as proteomics with functional validation could provide deeper insights into the molecular mechanisms underlying the processing stages. This knowledge would enable the optimization of processing techniques by modulating the expression of key genes and enzymes, ultimately enhancing black tea quality and boosting the development and economic benefits of the black tea industry.

## Materials and methods

### Tea preparation

**Fig. 7:**
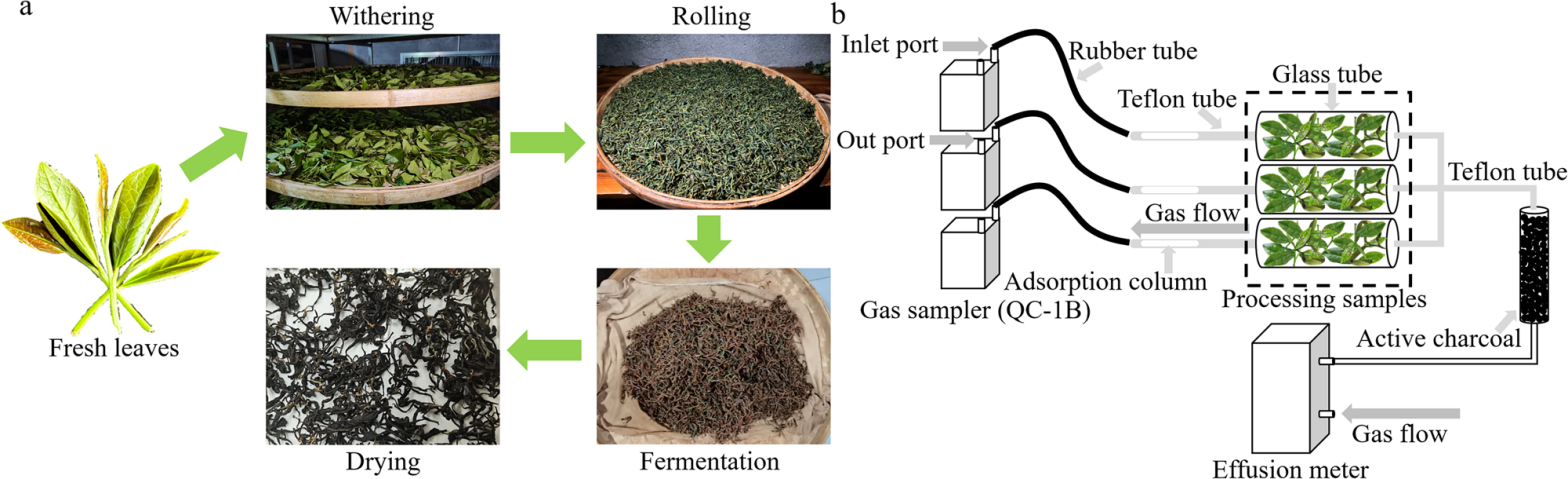
The traditional processing techniques of black tea and the working principle of the PPDH. **a** Black tea processing technology. **b** Collection device of volatiles.

One bud and two to three leaves from *Camellia sinensis* cv. Jinmudan were picked in the tea garden of Zhangdun Town, Jianyang District, Fujian Province (elevation 252.1 m, 27°25'33'' N, 118°28'41'' E) and processed using the traditional black tea manufacturing techniques (Fig. 7a). The process is as follows: The fresh tea leaves (FT) were naturally withered indoors until the leaf color turned dark green and the grassy aroma faded. They were then transferred to a specialized withering room (22 ± 1 °C, 45 ± 5% RH) to continue withering until the leaves became soft, the stems could be bent without breaking, and a fresh fragrance emerged, resulting in withered leaves (W). Next, the withered leaves were rolled. The rolling was considered adequate when the tea juice sufficiently exuded and adhered to the surface of the leaves without dripping. Immediately after rolling, the leaves were placed in a fermentation room at 27 ± 2 °C and 95% RH to ferment until 70% of the leaves turned red, indicating proper fermentation, thus obtaining the fermented leaves (F). The fermented leaves were then baked in a tea drying machine at 105 °C for 15 min, followed by further baking at 80 °C for 30 min. The tea was considered properly dried when it turned reddish-brown and could be easily crumbled by hand. This completed the preparation of the dried finished tea (D). The moisture content of tea samples at each stage is shown in Supplementary Table 6. A portion of the samples was immediately flash-frozen in liquid nitrogen and stored at −80 °C. Another portion was promptly placed into the apparatus shown in Fig. 7b for the collection of volatiles.

### Determination of non-volatile metabolites in tea

The freeze-dried tea samples were ground into powder, and 1 mL of 70% methanol (containing 3 ng/μL of 2′,7′-dichlorofluorescein as an internal standard) was added to 30 mg (±0.5 mg) of the tea powder. A 10 µL aliquot of the sample was further diluted 100-fold with 70% methanol and filtered through a 0.22 µm polyvinylidene fluoride (PVDF) filter (Millipore, Billerica, MA, USA) before analysis.

The analysis was performed using a Waters Acquity UPLC system coupled with a Waters photodiode array (PDA) detector and a SYNAPT G2-Si HDMS QTOF mass spectrometer (Waters, Manchester, UK), equipped with a Waters Acquity UPLC HSS T3 column (100 × 2.1 mm, 1.8 µm) for gradient elution. The mobile phase consisted of 0.1% formic acid in water (A) and 0.1% formic acid in acetonitrile (B), with an injection volume of 1 μL. The elution gradient was as follows: 0 min: 1% B; 2 min: 7% B; 13 min: 40% B; 17 min: 60% B, immediately increased to 99% B at 17 min and held for 5 min. The MS parameters were as follows: source temperature at 120 °C, desolvation temperature at 450 °C, collision energy at 4 eV, cone voltage at 40 eV, m/z range of 50–1200 Da, desolvation gas flow at 800 L/h, and cone gas flow at 50 L/h. Quality control (QC) samples were prepared by pooling equal amounts of all samples and were injected after every ten samples throughout the analytical run to monitor instrument performance.

The raw data were imported into Progenesis QI (Nonlinear Dynamics, Newcastle upon Tyne, UK), and data preprocessing was performed using default settings, with each sample normalized to the internal standard. The identification of metabolites was initially conducted using reliable standards, verifying them through the comparison of their retention times (RT) and MS/MS fragments. If there was no standard, the mass spectra information of metabolites was compared with HMDB, MassBank, ReSpect, Metlin, and KNApSAcK databases for preliminary identification, followed by verification through relevant literature. If necessary, collision-induced dissociation (CID) fragmentation of selected ions was performed to confirm structural assignments, and ultraviolet spectroscopy was used for identification as much as possible^30^.

### Real-time determination of tea volatiles

Volatiles were collected using the PPDH method^31^. Briefly, 15 g of tea samples from each processing stage were accurately weighed and placed into an assembled glass tube (Fig. 7b). The air purified through activated carbon was continuously introduced into the glass tube using a gas sampler (QC-1B), thereby drawing the volatiles released from the tea samples into a glass adsorption column containing 35 mg of Super-Q adsorbent (80–100 mesh) at a gas flow rate of 800 mL·min^-1^. The collection of volatiles lasted for 30 min, with each sample being replicated three times. After the collection, the volatiles were immediately eluted with 500 μL of chromatographic grade CH_2_Cl_2_ in three aliquots (200 μL, 200 μL, and 100 μL) into a 1.5 mL sample vial (Agilent) equipped with an insert. Then, 10 µL of 50 ppm ethyl decanoate was added as an internal standard.

The volatile components were analyzed using a SHIMADZU Nexis GC-2030 equipped with an autosampler (AOC-20i Plus) and a DB-5MS capillary column (30 m × 0.25 mm, 0.25 μm). High-purity helium (99.999%) was used as the carrier gas at a flow rate of 1.8 mL/min. The initial column temperature was maintained at 40 °C for 10 min, then increased at a rate of 3.5 °C/min to 210 °C without holding. Finally, the temperature was raised to 240 °C at a rate of 30 °C/min and held for 10 min. The ion source temperature was set at 230 °C, the interface temperature at 250 °C, with an electron energy of 70 eV, and a mass scan range of 40–400 m/z. Volatile peaks were identified by matching mass spectra with the National Institute of Standards and Technology (NIST 11) database (match factor ≥80). The chemical structures and names of the volatiles were determined using PubChem (https://pubchem.ncbi.nlm.nih.gov) and NIST (https://webbook.nist.gov/chemistry/cas-ser/), and odor descriptions were confirmed by consulting relevant literature. The relative content of volatiles was calculated using the internal standard method (Eq. (1)).

1 $${C}_{i}=A/B\times {D}_{i}$$

Note: *C*_*i*_ (ng) represents the relative content of volatile compounds; *A* represents content of internal standard; *B* represents peak area of internal standard; *D*_*i*_ represents the peak area of volatile compounds.

### Determination of volatile metabolites in tea

The freeze-dried tea samples were ground into powder, and 0.5 g of the tea powder was accurately weighed and mixed with 10 mL of boiling water, followed by the addition of 10 µL of 50 ppm ethyl decanoate. The headspace vial, equipped with an SPME injection handle, was placed in a 65 °C water bath for equilibration for 5 min. During this time, the fiber in the injection handle was not exposed. After equilibration, the fiber was exposed for 50 min to adsorb volatiles, and then the injection handle was immediately inserted into the GC-MS injection port for a 3-min desorption while the instrument was running. The GC-MS instrument and column specifications were as described in section 2.3, and manual injection was used. High-purity helium (99.999%) was used as the carrier gas at a flow rate of 1.8 mL/min. The initial column temperature was maintained at 40 °C for 3.5 min, then increased at a rate of 2 °C/min to 120 °C and held for 2 min. Finally, the temperature was raised to 230 °C at a rate of 10 °C/min and held for 2 min. The ion source temperature was set at 230 °C, the interface temperature at 250 °C, with an electron energy of 70 eV, and a mass scan range of 40-400 m/z.

2 $$y=13742175.38x-24960691.31$$

Note: x represents the ethyl decanoate volume; y represents the ethyl decanoate peak area. R^2^ = 0.99.

Volatile peaks were identified by matching mass spectra with the NIST11 database (match factor ≥80) and retention indices (RI, using n-alkanes C_7_-C_40_, with an error <20). Three samples of ethyl decanoate with different volumes were prepared and analyzed alongside the tea samples to establish a standard curve for ethyl decanoate volume and peak area (Eq. (2)). The content of volatiles was quantified using the internal standard method (Eq. (3)). The OAV of the volatile compounds is calculated by Eq. (4).

3 $${E}_{i}=({F}_{i}\times G\times {\rm{H}})/(I\times {\rm{M}})$$

Note: *E*_*i*_ (μg/kg) represents the content of volatile compounds; *F*_*i*_ represents the peak area of volatile compounds; *G* represents the volume of internal standard, which is calculated by Eq. (2); H represents the concentration of internal standard; *I* represents the peak area of internal standard; M represents tea sample weight.

4 $$O{{AV}}_{i}={E}_{i}/O{T}_{i}$$

Note: *E*_*i*_ represents the content of volatile compounds, which is calculated by Eq. (3); *OT*_*i*_ represents the odor threshold of volatile compounds.

### RNA sequencing and analysis

The total RNA was extracted using the RNAprep Pure Plant Kit (Tiangen, Beijing, China) according to the instructions provided by the manufacturer. RNA concentration and purity were measured using NanoDrop 2000 (Thermo Fisher Scientific, Wilmington, DE). RNA integrity was assessed using the RNA Nano 6000 Assay Kit of the Agilent Bioanalyzer 2100 system (Agilent Technologies, CA, USA). Sequencing libraries were generated using the Hieff NGS Ultima Dual-mode mRNA Library Prep Kit for Illumina (Yeasen Biotechnology (Shanghai) Co., Ltd.) following the manufacturer’s recommendations and index codes were added to attribute sequences to each sample, and 150 bp paired-end reads were generated on an Illumina NovaSeq platform. Clean reads were generated and aligned with the Huangdan genome (https://ngdc.cncb.ac.cn/gwh/Assembly/17995/show), and gene expression levels were estimated by fragments per kilobase of transcript per million fragments mapped. DESeq2 was used to perform differential expression analysis between the following groups: FT vs W, W vs F, and FT vs F. The resulting *P*-values were adjusted using the Benjamini and Hochberg’s approach for controlling the false discovery rate. Genes with an adjusted *P*-value < 0.01 & Fold Change≥2 found by DESeq2 were assigned as differentially expressed genes (DEGs). The sequencing data generated in this study are available in the NCBI SRA repository (BioProject ID: PRJNA1258312).

### qRT-PCR verification of RNA sequencing results

The cDNA templates for each sample were obtained using NovoScript® Plus All-in-one 1st Strand cDNA Synthesis SuperMix (gDNA Purge) (Nvoprotein, Shanghai, China), and qRT-PCR were performed using NovoStart® SYBR qPCR SuperMix Plus (E096; Nvoprotein, Shanghai, China). Primers were designed using Primer 5.0 and are listed in Supplementary Table 7. Each 20 μL reaction mixture was prepared independently as follows: 10 μL of fluorescent dye, 8 μL of RNase-Free Water, 0.5 μL each of forward and reverse primers, and 1 μL of cDNA template. The PCR reactions were carried out using the Quant Gene 9600 (TC-96/G/H(b)B; Bioer Technology, Hangzhou, China) real-time fluorescence quantitative PCR system with the following program: pre-denaturation at 95 °C for 1 min, followed by 40 cycles of denaturation at 95 °C for 20 s, annealing at 57 °C for 20 s, and extension at 72 °C for 30 s. Each sample was evaluated with three biological replicates and three technical replicates. The relative gene expression levels were calculated using the 2^−ΔΔCT^ method, with GAPDH as the reference gene.

### Statistics analysis

Quantitative data are presented as mean ± standard deviation (mean ± SD). Each sample was analyzed in triplicate. A one-way ANOVA and Tukey post hoc test were performed using SPSS 20.0, with a *P* value < 0.05 considered statistically significant. Multivariate statistical analysis was conducted using SIMCA 13.0, and correlation analysis, as well as the generation of heatmaps, network diagrams, volcano plots, rose diagrams, and bar charts, were performed using the R package.

## Supplementary information

Supplementary Data 1

Supplementary Data 2

Supplementary Data 3

Supplementary Data 4

Supplementary Data 5

Supplementary Data 6

Supplementary Data 7

Supplementary Data 8

Supplementary Data 9

Supplementary Data 10

Supplementary Data 11

Supplementary Data 12

Supplementary Data 13

Supplementary Data 14

Supplementary materials

## Data Availability

The authors declare that all pertinent data that support this study have been included within the paper. Raw data will be made available by corresponding authors upon request.

## Abbreviations

VTs: volatile terpenes
FADVs: fatty acid derivatives
AADVs: amino acid derivatives
UPLC - QTOF - MS: ultra performance liquid chromatography-quadrupole time-of-flight mass spectrometry
GC-MS: gas chromatography-mass spectrometry
HS-SPME: headspace solid-phase microextraction
PPDH: push - pull dynamic headspace collection
FT: fresh tea leaves
W: withered leaves
F: fermented leaves
D: dried finished tea
QC: quality control
RT: retention time
PVDF: polyvinylidene fluoride
CID: collision-induced dissociation
NIST: national institute of standards and technology
OAV: odor activity value
qRT-PCR: quantitative real-time polymerase chain reaction
PCA: principal component analysis
PLS-DA: partial least squares discriminant analysis
VIP: variable importance in projection
DEGs: differentially expressed genes
PPO: polyphenol oxidase
POD: peroxidase
CHS: chalcone synthase
CHI: chalcone isomerase
F3H: flavanone 3-hydroxylase
FLS: flavonol synthase
DFR: dihydroflavonol 4-reductase
LAR: leucoanthocyanidin reductase
ANR: anthocyanidin reductase
PKSB: type III polyketide synthase B
CYP75B2: flavonoid 3’-monooxygenase
AM: amylase
GT: glycosyltransferase
HPL: hydroperoxide lyase
LOX: lipoxygenase
ADH: alcohol dehydrogenase
PAAS: Phenylacetaldehyde synthase
PAR: Phenylacetaldehyde reductase
TPS: terpene synthase.
