# Supplementary material for: Metabolomics and transcriptomics reveal the quality formation mechanism during the processing of black tea
Source: NPJ Sci Food. 2025 Jul 9;9:131. doi: 10.1038/s41538-025-00488-7 (PMC12241338; doi:10.1038/s41538-025-00488-7)
Supplement: Supplementary file 15 — Supplementary materials [file 41538_2025_488_MOESM15_ESM.pdf]

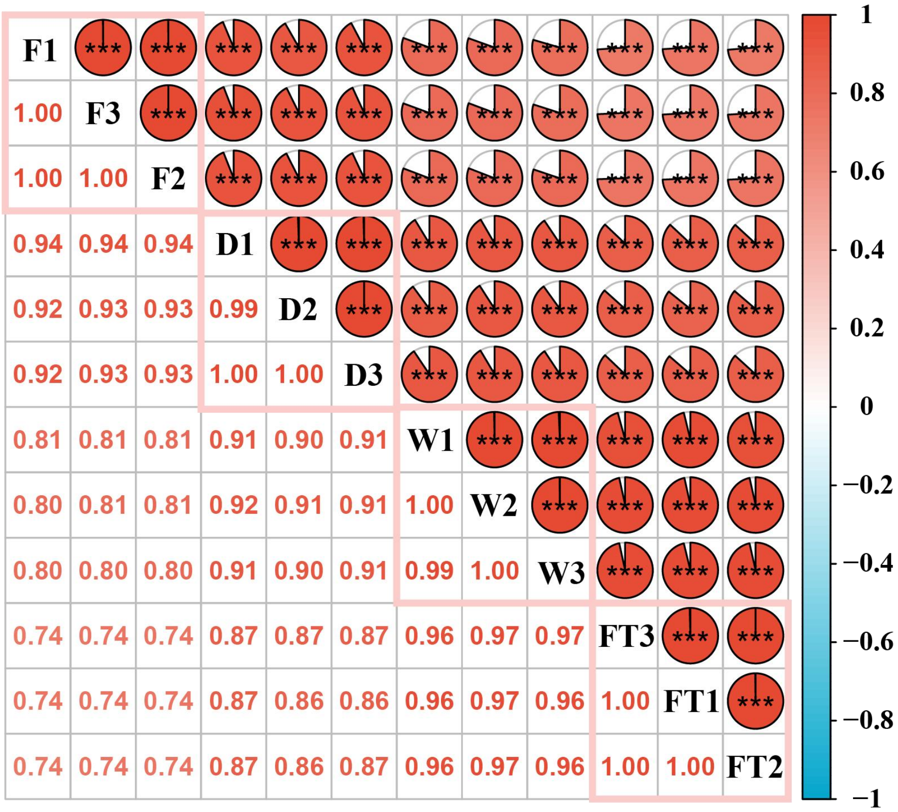

Supplementary Figure 1. Correlation heatmap between tea samples.

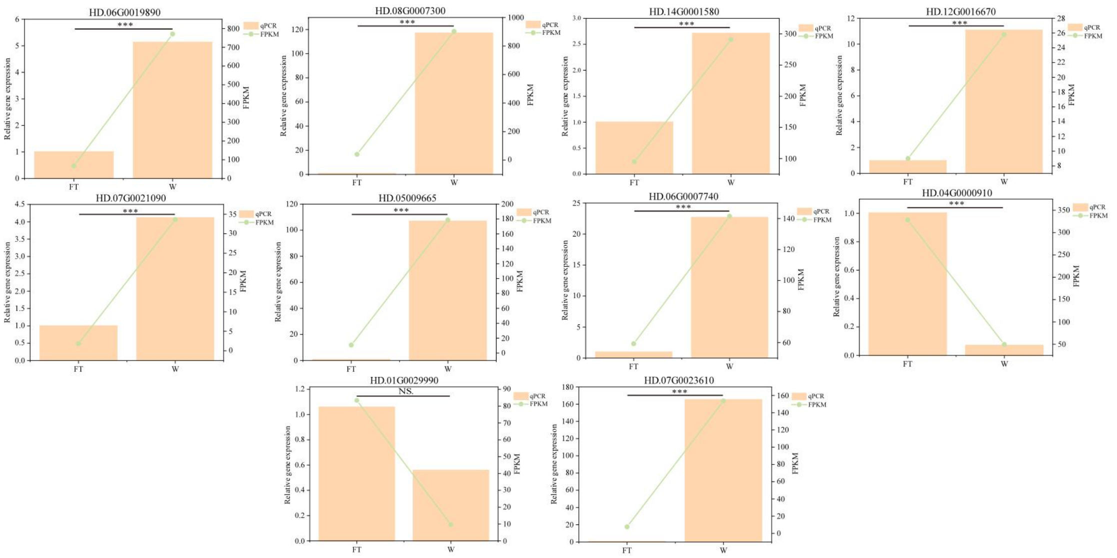

Supplementary Figure 2. Differential genes were validated using qRT-PCR. Significance of differences was tested by analysis of variance (ANOVA) and Tukey's test, with NS indicating no significant difference (P>0.05), * indicating significance at P<0.05, ** indicating significance at P<0.01, and *** indicating significance at P<0.001.

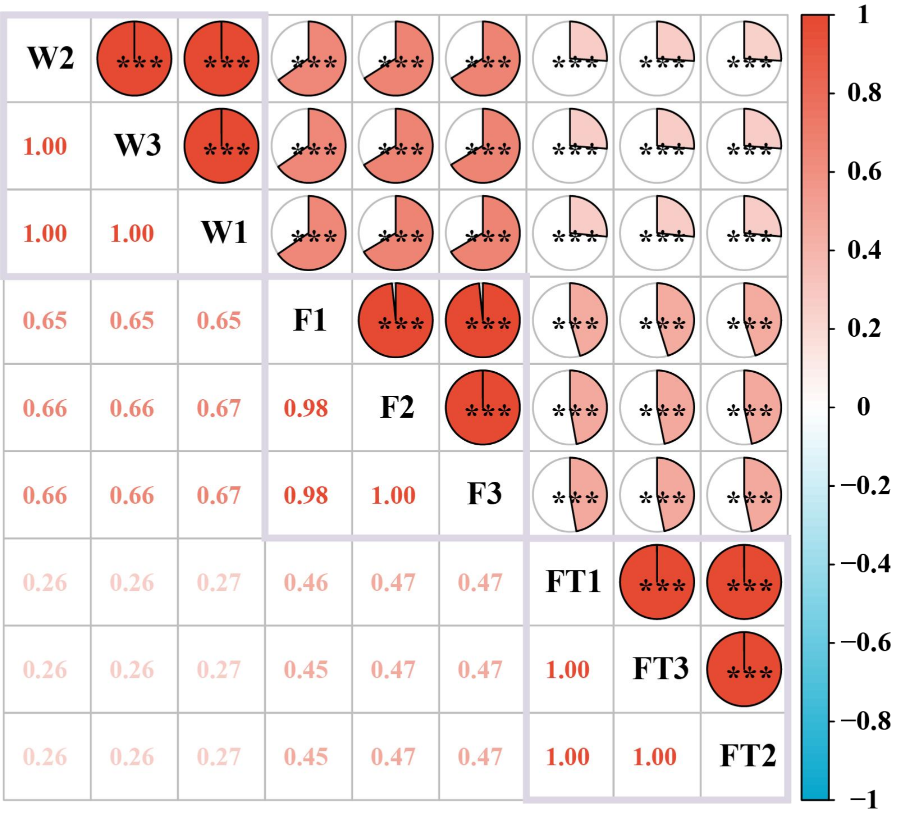

Supplementary Figure 3. Correlation heatmap between tea samples.

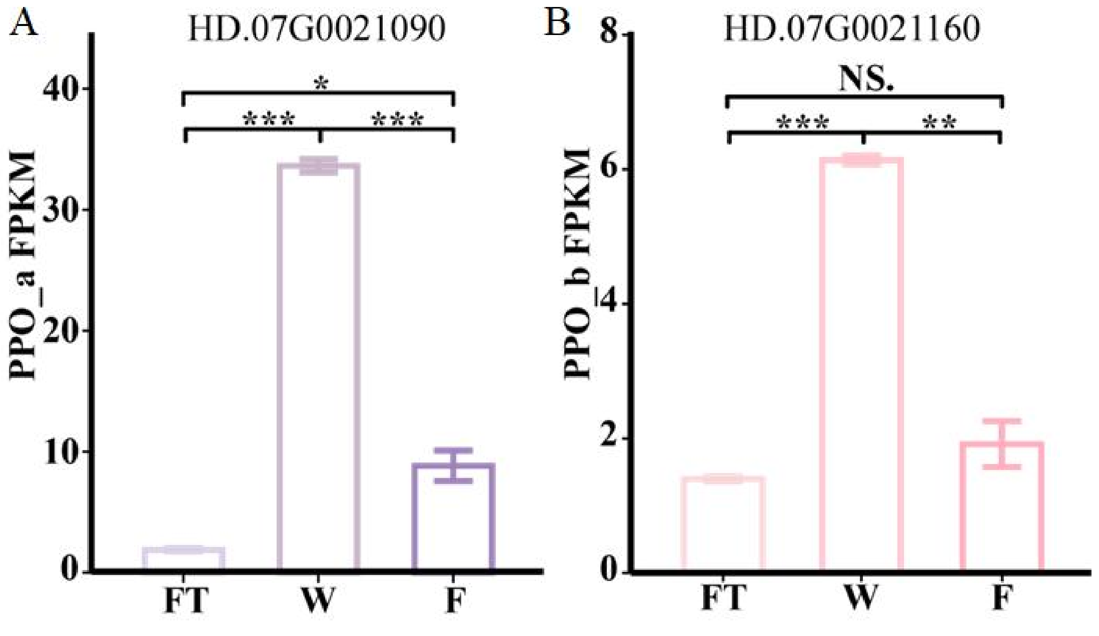

Supplementary Figure 4. The expression levels of PPO gene in different processing stages.

Supplementary Data 1. Non-volatile differential metabolites.

Supplementary Table 1. Non-volatile differential metabolites corresponding to the codename.

Metabolite	Class	Code
C-diglucosylapigenin	flavone glycosides	M1
kaempferol deoxyhexose-hexose-deoxyhexose isomer 1	flavone glycosides	M2

vitexin 2"-O-rhamnoside	flavone glycosides	M3
kaempferol 3-O-glucosyl rutinoside	flavone glycosides	M4
quercetin 3-O-glucosyl rutinoside	flavone glycosides	M5
kaempferol coumaroylrutinoside isomer 1	flavone glycosides	M6
kaempferol 3-(4"-(E)-p-coumaroylrobinobioside)-7-rhamnoside isomer 1	flavone glycosides	M7
kaempferol 3-O-galactoside	flavone glycosides	M8
kaempferol 3-O-galactosyl rutinoside	flavone glycosides	M9
capilliposide II isomer 2	flavone glycosides	M10
kaempferol 3-(4"-(E)-p-coumaroylrobinobioside)-7-rhamnoside isomer	flavone glycosides	M11
diglucopyranosyl trihydroxyflavanone	flavone glycosides	M12
Apigenin 7-arabinoside	flavone glycosides	M13
capilliposide I isomer 1	flavone glycosides	M14
kaempferol 3-O-rutinoside	flavonol glycosides	M15
quercetin 3-O-glucoside	flavonol glycosides	M16
apigenin 6-C-glucoside 8-C-arabinoside	flavonol glycosides	M17
5-Hydroxyferulic acid	phenolic acids	M18
chicoric acid isomer	phenolic acids	M19
3-p-coumaroylquinic acid	phenolic acids	M20
4-p-coumaroylquinic acid	phenolic acids	M21
Vanilpyruvic acid	phenolic acids	M22
chicoric acid isomer?	phenolic acids	M23
Procyanidin isomer	proanthocyanidins	M24
ECG-EGCG dimer isomer	proanthocyanidins	M25
procyanidin trimer isomer?	proanthocyanidins	M26
prodelphinidin B isomer 1	proanthocyanidins	M27
procyanidin B isomer	proanthocyanidins	M28
theanine	amino acids	M29
theanine isomer	amino acids	M30
glutathione	amino acids	M31
theaflavin	theaflavin	M32
theaflavin-3-gallate/isotheaflavin-3'-gallate/neotheaflavin-3-gallate	theaflavin	M33
sucrose	carbohydrates	M35
theasinensin B isomer 2	others	M34
linalool oxide primeveroside	others	M36
monogalloylglucose isomer 1	others	M37
unknown	others	M38
unknown	others	M39
unknown	others	M40
unknown	others	M41

unknown	others	M42
unknown	others	M43
unknown	others	M44

Supplementary Data 2. Real-time determination of volatiles during tea processing.

Supplementary Data 3. Volatiles in tea during processing.

Supplementary Table 2. The code name corresponds to the voaltiles.

Root	Class	Mark	Abundance	volatiles
aroma	alcohols	A1	21.42114721	Benzyl alcohol
aroma	alcohols	A2	82.98041221	2-Furanmethanol, 5-ethenyltetrahydro-.alpha.,.alpha.,5-trimethyl-, cis-
aroma	alcohols	A3	190.8554337	Linalool
aroma	alcohols	A4	19.98789831	Hotrienol
aroma	alcohols	A5	139.7089608	Phenylethyl Alcohol
aroma	alcohols	A6	24.63081756	(3R,6R)-2,2,6-Trimethyl-6-vinyltetrahydro-2H-pyran-3-ol
aroma	alcohols	A7	23.43501363	1,6-Octadien-3-ol, 3,7-dimethyl-, formate
aroma	alcohols	A8	2429.534039	2,6-Octadien-1-ol, 3,7-dimethyl-, (Z)-
aroma	alcohols	A9	51.36768209	1,6,10-Dodecatrien-3-ol, 3,7,11-trimethyl-, (E)-
aroma	alcohols	A10	15.87947983	Cedrol
aroma	alcohols	A11	24.40972726	3,7,11,15-Tetramethyl-2-hexadecen-1-ol
aroma	aldehydes	A12	10.10126808	Hexanal
aroma	aldehydes	A13	55.39747781	2-Hexenal, (E)-
aroma	aldehydes	A14	19.25020178	2,4-Hexadienal, (E,E)-
aroma	aldehydes	A15	42.76653395	Benzaldehyde
aroma	aldehydes	A16	148.3993428	Benzeneacetaldehyde
aroma	aldehydes	A17	32.44750673	Nonanal
aroma	aldehydes	A18	48.27779551	Decanal
aroma	aldehydes	A19	31.53424003	(Z)-3,7-dimethylocta-2,6-dienal
aroma	aldehydes	A20	71.09102899	2,6-Octadienal, 3,7-dimethyl-, (E)-
aroma	esters	A21	414.4656417	Methyl salicylate
aroma	esters	A22	12.23260393	cis-3-Hexenyl-.alpha.-methylbutyrate
aroma	esters	A23	36.36868402	1,2-Benzenedicarboxylic acid, bis(2-methylpropyl) ester
aroma	esters	A24	49.32477684	Hexanoic acid, 3-hexenyl ester, (Z)-
aroma	esters	A25	10.57400236	cis-3-Hexenyl cis-3-hexenoate
aroma	esters	A26	23.27460365	Hexadecanoic acid, methyl ester
aroma	esters	A27	14.43274911	3-Hexen-1-ol, benzoate, (Z)-
aroma	alkenes	A28	71.52645862	.beta.-Myrcene
aroma	alkenes	A29	38.02719842	trans-.beta.-Ocimene
aroma	alkenes	A30	50.56463587	1,3,6-Octatriene, 3,7-dimethyl-, (Z)-
aroma	alkenes	A31	49.23572824	.beta.-Ocimene
aroma	alkenes	A32	30.6110942	.alpha.-Farnesene
aroma	alkenes	A33	18.99054737	Neophytadiene

aroma	alkenes	A34	17.73119157	Naphthalene, 1,2,3,5,6,8a-hexahydro-4,7-dimethyl-1-(1-methylethyl)-, (1S-cis)-/isoledene
aroma	alkanes	A35	5.779638872	Heneicosane
aroma	alkanes	A36	4.401942011	Heptadecane, 2,6,10,15-tetramethyl-
aroma	ketones	A37	34.86598965	2-Cyclopenten-1-one, 3-methyl-2-(2-pentenyl)-, (Z)-
aroma	ketones	A38	12.0111487	5,9-Undecadien-2-one, 6,10-dimethyl-, (E)-
aroma	ketones	A39	16.31554131	trans-.beta.-Ionone
aroma	ketones	A40	11.87835715	2H-Pyran-2-one, tetrahydro-6-pentyl-
aroma	ketones	A41	9.334689087	Acetophenone
aroma	fatty acids	A42	12.98674949	Tetradecanoic acid
aroma	fatty acids	A43	29.40106953	n-Hexadecanoic acid
aroma	aromatic compounds	A44	54.47189967	Benzyl nitrile
aroma	aromatic compounds	A45	9.323206284	Indole
aroma	others	A46	405.4273699	Caffeine

Supplementary Table 3. Characteristic volatiles.

Volatiles	Odor description	OT (μg/kg)	OAVs			
			FT	W	F	D
Linalool	Floral, sweet, grape-like	5	56.06	103.5	85.26	38.17
nerol	floral,woody	49	30.06	62.32	101.23	49.58
Phenylethyl Alcohol	Floral, rose-like	140	0.3	0.29	0.44	1
.beta.-Myrcene	fruity,sweet	15	0.7	2.96	30.05	4.77
Methyl salicylate	Minty, fresh, sweet	40	6.55	12.92	24.06	10.36
Indole	Floral	11	24.69	3.09	1.59	0.85
Benzeneacetaldehyde	Clean aroma, rose-like, floral and chocolate-like smell	5.2	0	0	9.65	28.54
2-Hexenal, (E)-	Green, fresh, fruity	17	1.45	10.84	8.12	3.26

Supplementary Table 4. The basic information of transcriptome data.

#SampleID	Clean Reads	Clean Bases	GC(%)	N(%)	Q20(%)	Q30(%)
FT-1	22498968	6718434088	44.96	0	99.69	98.4
FT-2	23395830	6985117694	44.93	0	99.74	98.63
FT-3	23946588	7150672437	45.06	0	99.73	98.57
W1	24036166	7175322381	44.98	0	99.7	98.44
W2	23027054	6873214098	44.97	0	99.67	98.39
W3	22651556	6758059763	44.91	0	99.7	98.45

F1	24998243	7468794701	44.77	0.04	98.3	95.36
F2	23655388	7071962581	44.68	0	99.73	98.59
F3	22213137	6642966021	44.65	0	99.72	98.51

Supplementary Table 5. Comparison efficiency between reads and reference genome of each sample.

Sample	Total Reads	Mapped Reads	Uniq Mapped Reads	Multiple Map Reads	Reads Map to '+'	Reads Map to '-'
FT-1	44,997,936	42,516,433 (94.49%)	40,931,409 (90.96%)	1,585,024 (3.52%)	22,383,769 (49.74%)	22,378,058 (49.73%)
FT-2	46,791,660	44,232,679 (94.53%)	42,564,581 (90.97%)	1,668,098 (3.56%)	23,288,965 (49.77%)	23,280,655 (49.75%)
FT-3	47,893,176	45,309,982 (94.61%)	43,600,189 (91.04%)	1,709,793 (3.57%)	23,865,257 (49.83%)	23,852,759 (49.80%)
W1	48,072,332	45,603,894 (94.87%)	43,279,385 (90.03%)	2,324,509 (4.84%)	24,602,057 (51.18%)	24,593,444 (51.16%)
W2	46,054,108	43,745,341 (94.99%)	41,452,425 (90.01%)	2,292,916 (4.98%)	23,655,728 (51.37%)	23,651,915 (51.36%)
W3	45,303,112	43,067,176 (95.06%)	40,842,341 (90.15%)	2,224,835 (4.91%)	23,263,573 (51.35%)	23,264,877 (51.35%)
F1	49,996,486	43,803,561 (87.61%)	42,040,281 (84.09%)	1,763,280 (3.53%)	23,226,582 (46.46%)	23,251,276 (46.51%)
F2	47,310,776	42,374,206 (89.57%)	40,619,967 (85.86%)	1,754,239 (3.71%)	22,503,954 (47.57%)	22,506,790 (47.57%)
F3	44,426,274	39,474,987 (88.86%)	37,862,183 (85.22%)	1,612,804 (3.63%)	20,939,814 (47.13%)	20,944,084 (47.14%)

Supplementary Data 4. Genes expressed in at least one sample.

Supplementary Data 5. Common differential genes among diffrenent comparison groups.

Supplementary Data 6. 60 genes involved in differential accumulation of flavonoids.

Supplementary Data 7. Details of AM genes.

Supplementary Data 8. Details of GT genes.

Supplementary Data 9. Details of the TPS genes.

Supplementary Data 10. Details of the LOX genes.

Supplementary Data 11. Details of the HPL genes.

Supplementary Data 12. Details of the ADH genes.

Supplementary Data 13. Details of the PAAS genes.

Supplementary Data 14. Details of the PAR genes.

Supplementary Table 6. Moisture content of tea at each processing stage.

Samples	Moisture content
FT1	79.28%
FT2	78.79%
FT3	78.31%
mean±SD	0.7879±0.0049a
W1	65.54%

W2	66.43%
W3	67.88%
mean±SD	0.6662±0.0118b
F1	61.33%
F2	65.8%
F3	61.17%
mean±SD	0.6277±0.0263b
D1	9.86%
D2	9.29%
D3	8.62%
mean±SD	0.0926±0.0062c

Note: Different letters indicate significant differences (P<0.05).

Supplementary Table 7. Primers used for qRT-PCR.

Gene ID	Primers (5’-3’)
HD.06G0019890	F: AGAAGCATGGCCTCAAGGTC
	R：GTGTACGTCCTTTGAGGGCA
HD.08G0007300	F: TCCCGGAATTGGAGCCTTTC
	R: CAACCACCGCCTTCTTTTCG
HD.04G0000910	F: TGTCGAAGCTTGATCGCCTT
	R: CCACAACTCAGGAATCCCCC
HD.06G0007740	F: ATCCTGCCAAGCGTCAGTTT
	R: CAAGGAAGAAAGGCCCGTCA
HD.05009665	F: ATCCATGTGTGGGCCAGATG
	R: CGCCTAGTCACTGATCGCAT
HD.01G0029990	F: ATGGTTCCTGGTGGCATCAG
	R: GGATGGGAGCACTAGACTGC
HD.07G0021090	F: ACAAGGCGGTGAGAGTGATG
	R: ATCCTCAGCTCCCAACTCCT
HD.12G0016670	F: CGGCTCATCTACACGGCTAG
	R: CACAAGTGCACAAGCCAGAC
HD.07G0023610	F: TCAACGACTTCCAGAGTGCA
	R: ACCACCTTGCTTCCACAAGT
HD.14G0001580	F: GTACCGAGTTGTCCATGGCA
	R: GTTTTTCACCCGGTCAAGCC
